# Otolith Morphological Characteristics of Major Schizothoracinae Fish Species in Tibet and Genus-Level Morphological Discrimination

**DOI:** 10.3390/ani16182883

**Published:** 2026-09-13

**Authors:** Yunfei Wang, Yang Zhou, Liuxuan Qiao, Haoran Zhang, Lijing Long, Jian Su, Chaowei Zhou, Zhaofang Han, Haiping Liu

**Affiliations:** 1 College of Fisheries, Southwest University, Chongqing 400715, China; 2 Western (Chongqing) Science Center for Germplasm Creation, Chongqing 400050, China; 3 Key Laboratory of Freshwater Fish Reproduction and Development, Ministry of Education, Chongqing 400715, China; 4 Institutes of Fisheries Science, Neijiang Academy of Agricultural Sciences, Neijiang 641000, China

**Keywords:** cyprinidae, geometric morphometrics, phenotypic differentiation, extreme environment, shape indices, Elliptic Fourier Analysis, Qinghai–Xizang Plateau

## Abstract

Schizothoracinae represent an endemic and dominant teleost group in the Qinghai–Xizang Plateau and adjacent regions. Despite their rich species diversity, closely related Schizothoracinae species exhibit remarkable external morphological similarity, which hinders traditional morphological discrimination. In this study, lapillus otoliths from 360 individuals representing 12 Schizothoracinae species collected across major Tibetan water systems were systematically examined. We evaluated and compared the performance of traditional scalar-based shape indices and Elliptic Fourier Analysis (EFA, 60 normalized harmonic coefficients following size-allometry correction) in otolith contour quantification and species identification. Statistical analyses including one-way ANOVA, principal component analysis (PCA), non-linear manifold learning (t-SNE and UMAP), and linear discriminant analysis (LDA) were executed. One-way ANOVA revealed highly significant differences among all 12 species across all shape indices and Fourier coefficients. The overall LDA classification accuracy was 35.3% for the shape index method, whereas EFA achieved a higher overall accuracy of 68.3%, representing a 33.0 percentage point improvement. Under EFA, *Schizothorax lhasaensis* (93.3%) and *Schizothorax nukiangensis* (90.0%) demonstrated the highest single-species discrimination success. Interspecific otolith morphological divergence was substantially higher in the riverine genus *Schizothorax* than in lacustrine genera (*Gymnocypris* and *Schizopygopsis*), the latter two of which show extensive morphological overlap consistent with their low genomic differentiation. Fishes of *Gymnocypris* displayed significantly higher otolith roundness (*p* < 0.05), which correlates with their adaptation to lacustrine or sluggish water habitats. Our findings demonstrate that EFA effectively captures multi-scale otolith contour harmonics, serving as a valuable morphometric approach for Schizothoracinae species identification and morphological discrimination, although challenges remain for species with incomplete lineage sorting. The morphometric database established herein provides valuable morphological baselines for the conservation and management of endemic QXP fish resources.

## Simple Summary

Identifying native fish species on the Qinghai–Xizang Plateau is challenging because many closely related species look remarkably similar. This study evaluated the efficacy of two-dimensional otolith shape analysis for distinguishing twelve native high-altitude fish species across four genera in Tibet. Advanced mathematical contour analysis (Elliptic Fourier Analysis combined with LDA) successfully improved the overall morphological discrimination accuracy from 35% to 68%. Notably, river-dwelling fishes of *Schizothorax* possessed elongated otoliths with pointed ends, achieving discrimination rates up to 93%. In contrast, lake-dwelling fishes of *Gymnocypris* and *Schizopygopsis* exhibited rounded otoliths with extensive shape overlap. This morphological similarity reflects their low genetic divergence and shared adaptation to calm lake habitats. Ultimately, this research provides a valuable morphometric baseline to complement existing molecular tools for monitoring fish populations and conserving vulnerable freshwater ecosystems.

## 1. Introduction

The subfamily Schizothoracinae (family Cyprinidae) is a unique teleost group widely distributed across the Qinghai–Xizang Plateau (QXP) and its peripheral drainage basins. Originating from ancestral Barbinae fishes alongside the continuous stepwise uplift of the QXP, Schizothoracinae have undergone extraordinary adaptive radiation in response to extreme high-altitude environments characterized by low temperatures, hypoxia, and intense ultraviolet radiation [1,2]. Occupying key ecological niches in high-altitude freshwater ecosystems, this clade currently encompasses more than ten genera and nearly one hundred recognized species, constituting the core component of the fish fauna in Western China [3]. Due to the extremely high-altitude environment, native Tibetan fishes generally exhibit unique life-history traits, such as slow growth rates, delayed sexual maturity, and low fecundity [4]. Consequently, their fragile populations are highly susceptible to dual pressures from global climate change and increasing anthropogenic disturbances (e.g., dam construction, habitat fragmentation, and overfishing), underscoring the urgent need for precise species identification and continuous biodiversity monitoring in the QXP [5].

However, the taxonomy and systematics of Schizothoracinae have long encountered persistent difficulties. High morphological similarity, conservative external meristic characters, and overlapping diagnostic traits severely hinder precise species identification, particularly among closely related sympatric species [2]. Driven by climate change and intensified anthropogenic activities, wild populations of several Schizothoracinae species have suffered marked declines, highlighting an urgent need for accurate and efficient species identification tools to support biodiversity conservation and fisheries management. Consequently, developing objective, quantitative taxonomic methodologies remains of paramount importance. While molecular tools are widely used, otolith morphometrics is intended to complement, rather than replace, these existing molecular identification methods by providing crucial phenotypic and life-history perspectives.

Otoliths—calcified structures situated in the inner ear membranous labyrinth of teleost fishes—possess strong species specificity and high morphological stability, rendering them valuable tools in fish taxonomy, population discrimination, and phylogenetics [6,7]. Growing continuously throughout a fish’s life cycle while remaining metabolically inert, otoliths permanently record age, growth, life history, and environmental signatures [7].In recent decades, otolith morphometrics has been broadly applied across diverse fish taxa [8,9,10]. Methodologically, Tracey et al. [11] introduced Elliptic Fourier Analysis (EFA) into otolith morphometrics for stock identification of striped trumpeter (*Latris lineata*), achieving an 87% classification success. Afanasyev et al. [12] systematically reviewed geometric morphometrics in fish identification, pointing out the broad prospects of EFA. Ponton [13] compared morphometric techniques in clupeid fishes and found geometric methods highly efficient for sagittal otolith comparison. Mesa et al. [14] evaluated LDA, Random Forest, and K-Nearest Neighbors in icefishes (Channichthyidae), demonstrating the utility of multi-classifier evaluations. Among otolith shape analysis approaches, traditional scalar shape indices and Elliptic Fourier Analysis represent two prominent frameworks [15,16].

In Schizothoracinae, otolith morphometrics has seen preliminary applications. Wang et al. [17] analyzed three *Schizothorax* species in the Yarkand River, showing that traditional shape parameters explained 81.24% of variation with an 81.65% classification accuracy, which reached 100% upon incorporating 77 Fourier harmonics. Zhou et al. [18] discriminated three geographical stocks of *Schizothorax grahami* using shape indices, obtaining a 59.7% success rate. Additional successful applications of otolith EFA include mudskippers [19], *Scomber* spp. [20], juvenile cyprinids [21], and flatfishes [22].

In the present study, lapillus otoliths from 360 individuals belonging to 12 Schizothoracinae species (representing four genera) across Tibetan river systems and plateau lakes were systematically examined. By employing both traditional shape indices and size-allometry-corrected Elliptic Fourier Analysis, we aimed to: (1) evaluate and compare the efficacy of the two shape quantification methods in species discrimination; (2) reveal the genus-level otolith morphological divergence patterns among Schizothoracinae; (3) elucidate the morphological discrimination and biological limitations of otolith morphology under the evolutionary context of the Qinghai–Xizang Plateau.

## 2. Materials and Methods

### 2.1. Study Area and Sample Collection

**Figure 1:**
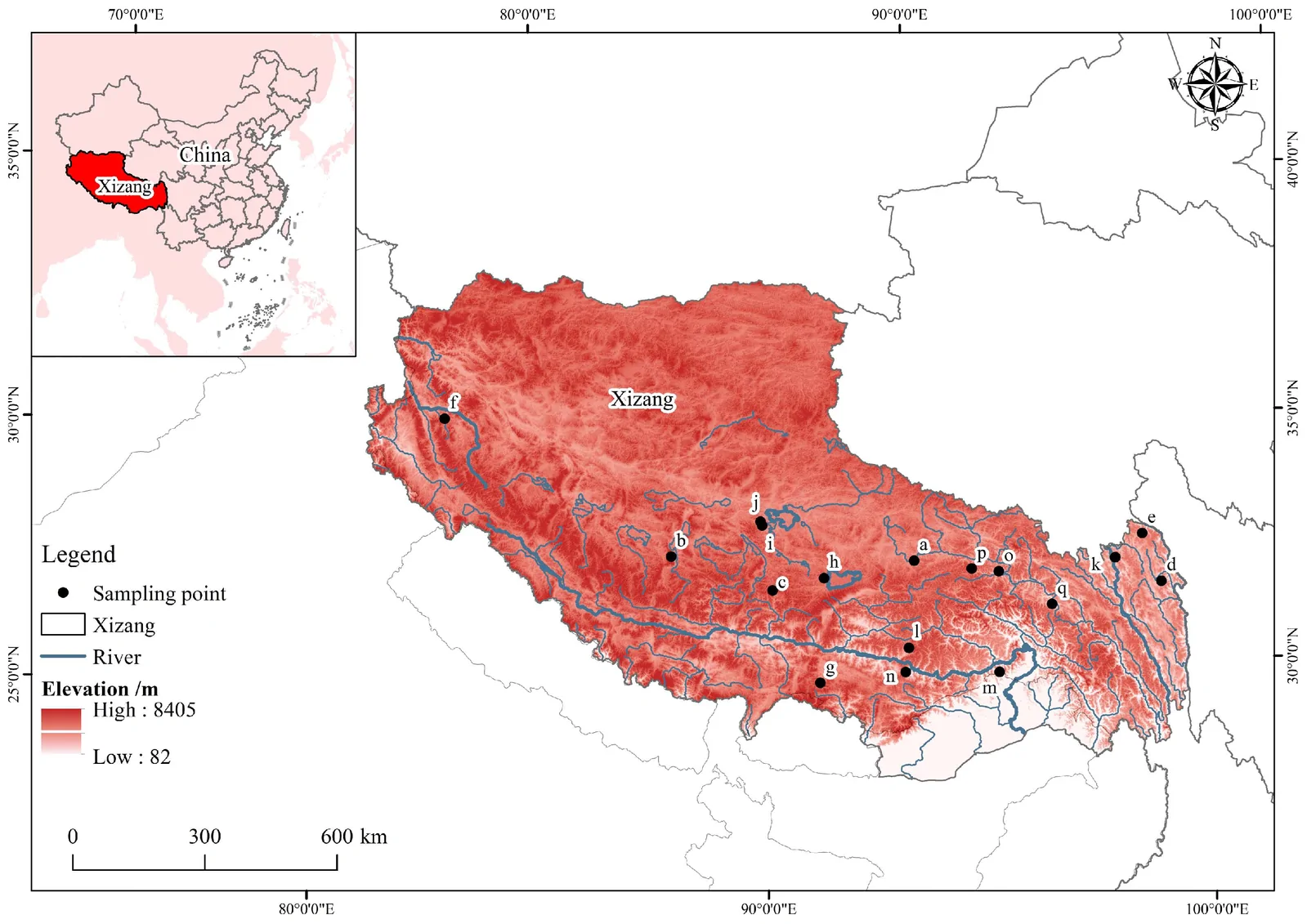
Sampling locations of the studied Schizothoracinae species across the Qinghai–Xizang Plateau, with the sampling point numbers explained in Table 1.

In 2024, a total of 360 adult specimens representing 12 Schizothoracinae species (30 individuals per species) were collected across major river systems (Yarlung Zangbo River, Nujiang River, Jinshajang River) and plateau lakes (Siling Co, Namtso, Yamdrok Younco) in the hinterland of the Qinghai–Xizang Plateau using multi-mesh gill nets (mesh sizes ranging from 2.0 to 6.0 cm; 30 m long, 1.5 m deep) and trap nets (5 m in length, 30 × 30 cm rectangular frames, with 0.5 cm mesh size). The fishing gears were typically set in the evening and retrieved the following morning, with an average operating time of approximately 12–14 h per deployment. Sampling locations are illustrated in Figure 1, and detailed sample metadata are summarized in Table 1. In the field, total length (TL, 0.1 cm) and body weight (W, 0.1 g) were recorded for each specimen. Left and right lapillus otoliths were dissected using fine forceps, cleaned of adhering tissues in an ultrasonic cleaner using distilled water, air-dried, and stored in labeled 1.5 mL microcentrifuge tubes. Ethical review and approval were waived for this study. According to Article 2 of the Chinese Regulations for the Administration of Laboratory Animals, laboratory animals are defined as those artificially bred and reared under controlled conditions for scientific use. The wild-caught fish utilized in this field survey do not fall within that category, and their collection was conducted strictly under the relevant fisheries legislation and an official Special Fishing Permit issued by the Department of Agriculture and Rural Affairs of the Tibet Autonomous Region. The specimens were intentionally captured during a scientific field survey using gill nets and trap nets. Some individuals experienced capture-related accidental mortality (e.g., through exhaustion or asphyxiation) and were found dead upon retrieval following capture. Otoliths were collected exclusively from these specimens, and only after death had been thoroughly verified.

### 2.2. Otolith Image Acquisition and Contour Extraction

**Figure 2:**
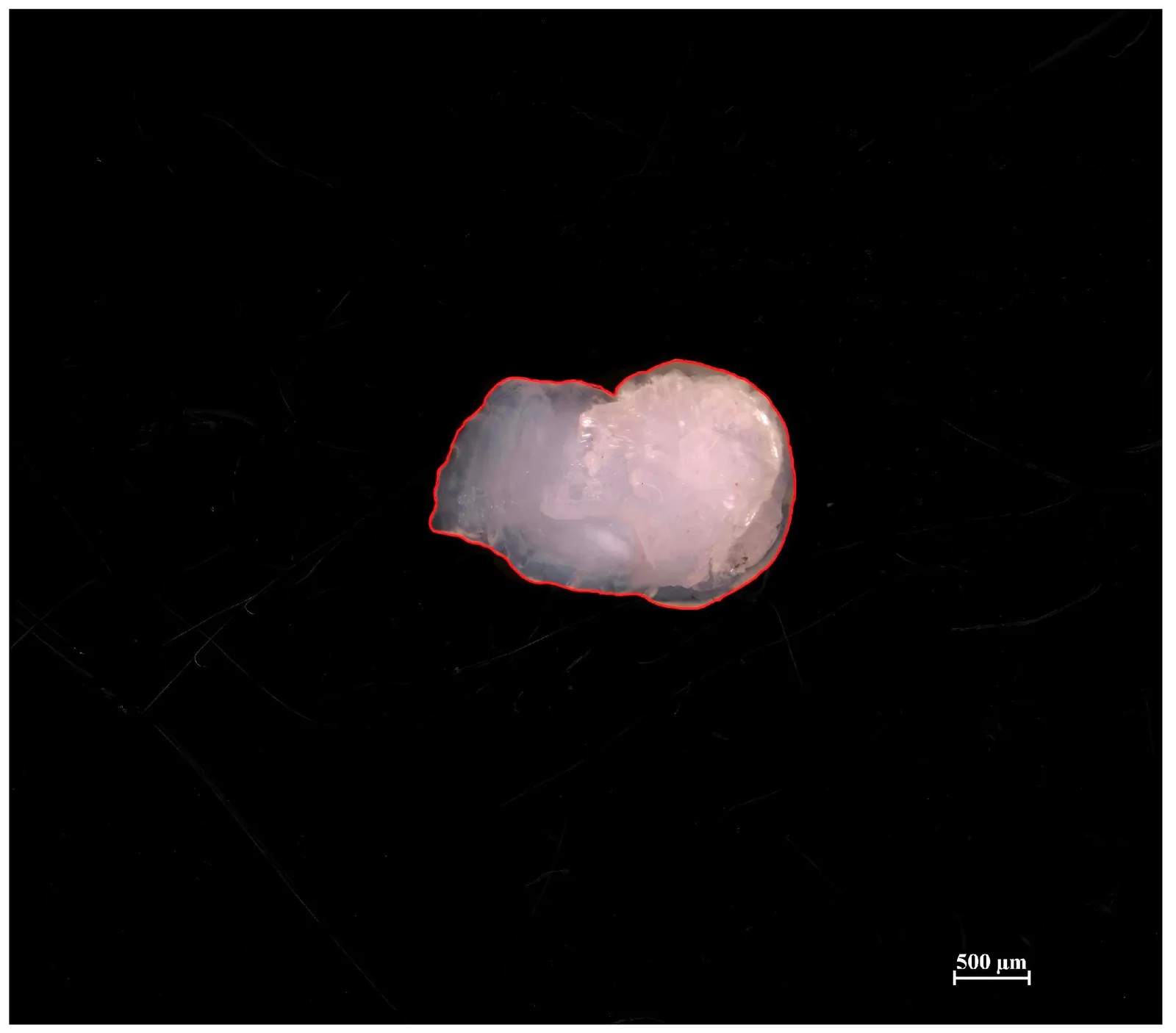
Diagram of otolith placement and contour extraction.

High-resolution digital images of lapillus otoliths were captured using a stereomicroscope (Motic SM171, Motic China Group Co., Ltd., Xiamen, China) equipped with a digital camera (Moticam S6, Motic China Group Co., Ltd., Xiamen, China). All images were taken under identical optical magnification, focal distance, and standardized illumination. The sulcus acusticus was oriented upward and the rostrum pointing to the left (Figure 2).

To overcome the limitations of single-threshold edge detection and path-encoding incompatibilities in traditional packages (e.g., shapeR), we developed an image processing framework using Python (version 3.12.8) and OpenCV (version 5.0.0). Images were smoothed using a Gaussian filter, and optimal binary segmentation was achieved via Otsu’s maximum inter-class variance thresholding. Morphological closing operations were applied to eliminate edge noise and fill internal vacuities. Outer closed contours were extracted, translated to their geometric centroid (0, 0), and normalized by cumulative arc length. Contours were resampled into *n*= 512 equidistant boundary coordinates, resolving multi-value mapping issues inherent to conventional polar coordinates. A resolution of 512 resampled points was selected to optimize the computational efficiency of the Fast Fourier Transform algorithms while providing sufficient spatial resolution without introducing pixel-level noise.

### 2.3. Shape Index Computation

**Figure 3:**
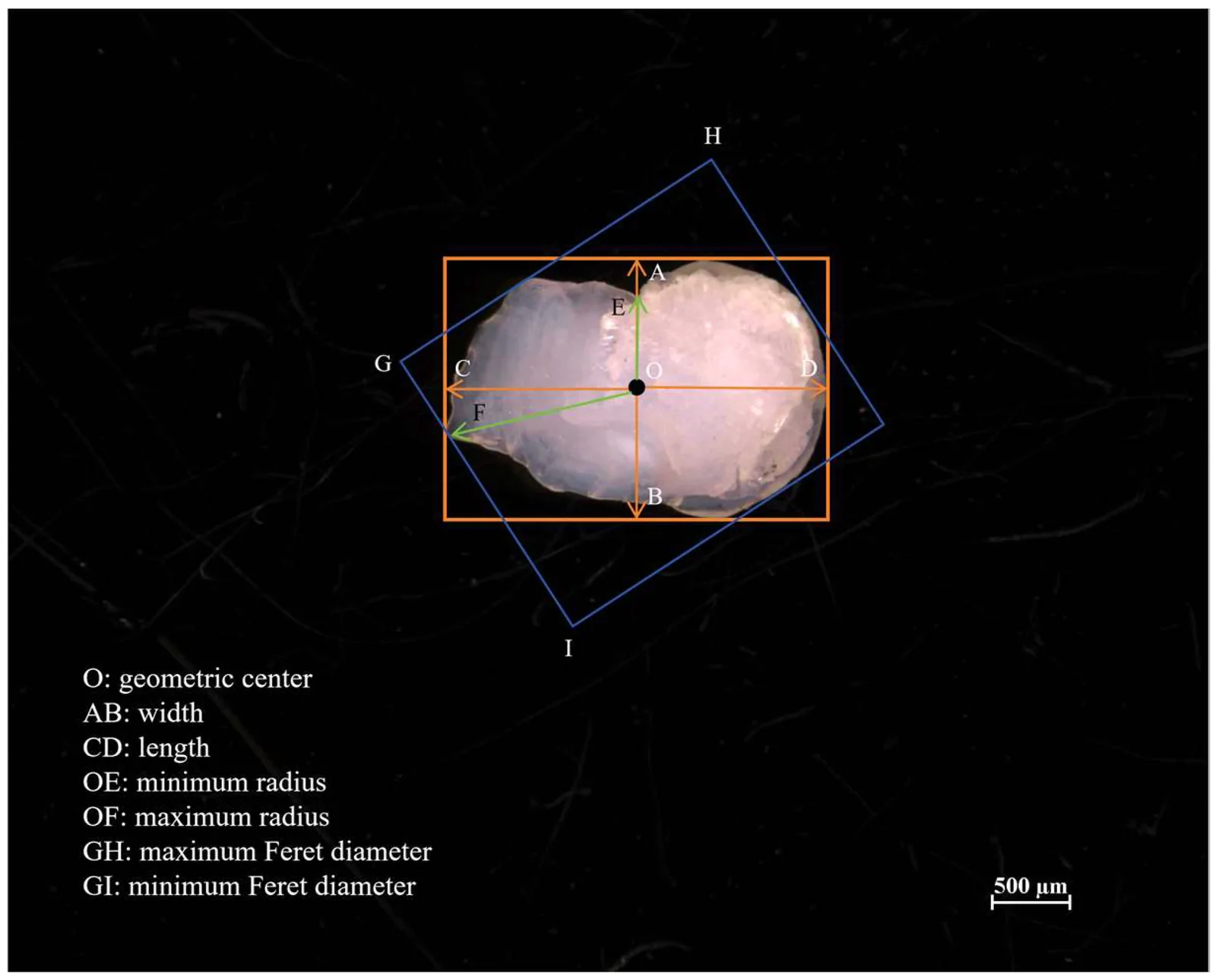
Measurement illustration of otolith morphometric parameters.

Eight basic morphometric parameters were measured: area (A), perimeter (P), Otolith length (FL), Otolith width (FW), Maximum Feret diameter (Fmax), Minimum Feret diameter (Fmin), Maximum radius (Rmax), and Minimum radius (Rmin) using Image-Pro Plus 6.0 (Figure 3). From these, eight dimensionless shape indices were derived (Table 2):

The 8 otolith shape indices were analyzed. One-way ANOVA, principal component analysis, t-SNE dimensionality reduction, and linear discriminant analysis were used to evaluate the differentiation characteristics and discriminant performance of otolith shape among species.

### 2.4. Elliptic Fourier Analysis

According to the algorithm of Kuhl and Giardina [15], Elliptic Fourier Analysis was performed on the resampled 512-point contours. The first 15 harmonics (*n* = 15) were extracted, yielding 60 normalized Fourier coefficients. Coefficients were aligned, rotated, and scaled relative to the major axis of the first harmonic ellipse, rendering descriptors invariant to otolith size, orientation, and starting point. Inverse Elliptic Fourier Analysis (IEFA) was executed to mathematically reconstruct the mean otolith contours for all 12 species.

### 2.5. Size-Allometry Growth Correction

To remove the confounding effect of fish body size on otolith shape, all 60 Fourier coefficients were adjusted using common allometric slope subtraction [7,23]:

C_adj = C − b × (TL − TL_mean)

where C is the raw Fourier coefficient, C_adj is the size-corrected coefficient, b is the within-group linear regression slope of C against fish length TL, and TL_mean is the overall mean body length of all samples (30.42 cm).

### 2.6. Multivariate Statistical Analysis

Both standardized shape indices and size-corrected Fourier coefficients were Z-score normalized prior to statistical modeling. One-way ANOVA and Tukey’s HSD post hoc tests were performed to test interspecific differences. Principal component analysis (PCA) was conducted to extract the main orthogonal axes of shape variation. Non-linear manifold learning algorithms—t-Distributed Stochastic Neighbor Embedding (t-SNE, perplexity = 30) and Uniform Manifold Approximation and Projection (UMAP, n_neighbors = 25)—were employed to visualize local clustering. Linear discriminant analysis (LDA) with Leave-One-Out Cross-Validation (LOOCV) was constructed to calculate classification accuracy and generate confusion matrices with 95% confidence ellipses.

## 3. Results

### 3.1. Shape Index Approach

#### 3.1.1. One-Way ANOVA

One-way ANOVA results showed that all 12 species exhibited significant differences in shape indices (Table 3), demonstrating that otolith morphology possesses highly significant statistical differentiation among species.

Feret ratio (F = 21.624) and aspect ratio (F = 19.054) exhibited the highest F-values among all indices, being the two shape indices contributing most to distinguishing the 12 Schizothoracinae species. Both indices reflect the length-to-width ratio of the otolith’s bounding rectangle. This indicates that elongation along the major-minor axes is the primary dimension of interspecific differentiation; form factor (F = 18.301) and ellipticity (F = 18.111) ranked second, mainly measuring the relationship between otolith area and perimeter as well as the degree of approaching an ellipse, reflecting the development degree of otolith marginal indentations/projections (Rostrum/Antirostrum), serving as the second largest discriminating trait; circularity (F = 17.448) and roundness (F = 17.448) had relatively lower F-values, but still reached extremely significant levels.

At the species level, *Schizothorax lhasaensis* exhibited significantly higher ellipticity (0.201 ± 0.022) than other species, Feret ratio (1.532 ± 0.067), and aspect ratio (1.506 ± 0.068) (*p* < 0.05), indicating that its otolith shape is the most elongated with prominent major axis extension and the highest degree of marginal specialization; *Ptychobarbus dipogon* was at the lowest level in ellipticity (0.109 ± 0.036), Feret ratio (1.279 ± 0.073), and aspect ratio (1.247 ± 0.091), with otoliths being the most rounded, short, and wide; *Gymnocypris* selincuoensis was significantly lower than other species in form factor (0.727 ± 0.069) (*p* < 0.05), suggesting that its otolith margin is the most smooth and regular.

At the genus level, interspecific differentiation in the genus *Schizothorax* was the most significant (generally higher F-values), among which *Schizothorax lhasaensis* exhibited extremely high specificity and statistical significance across multiple indices; only a few indices exhibited significant differences among the four *Schizopygopsis* species, indicating that otolith morphology within this genus is relatively conservative; the form factor of the three *Gymnocypris* species was significantly lower than that of other genera (*p* < 0.05), indicating that otolith morphology of *Gymnocypris* is the most rounded and smooth.

**Figure 4:**
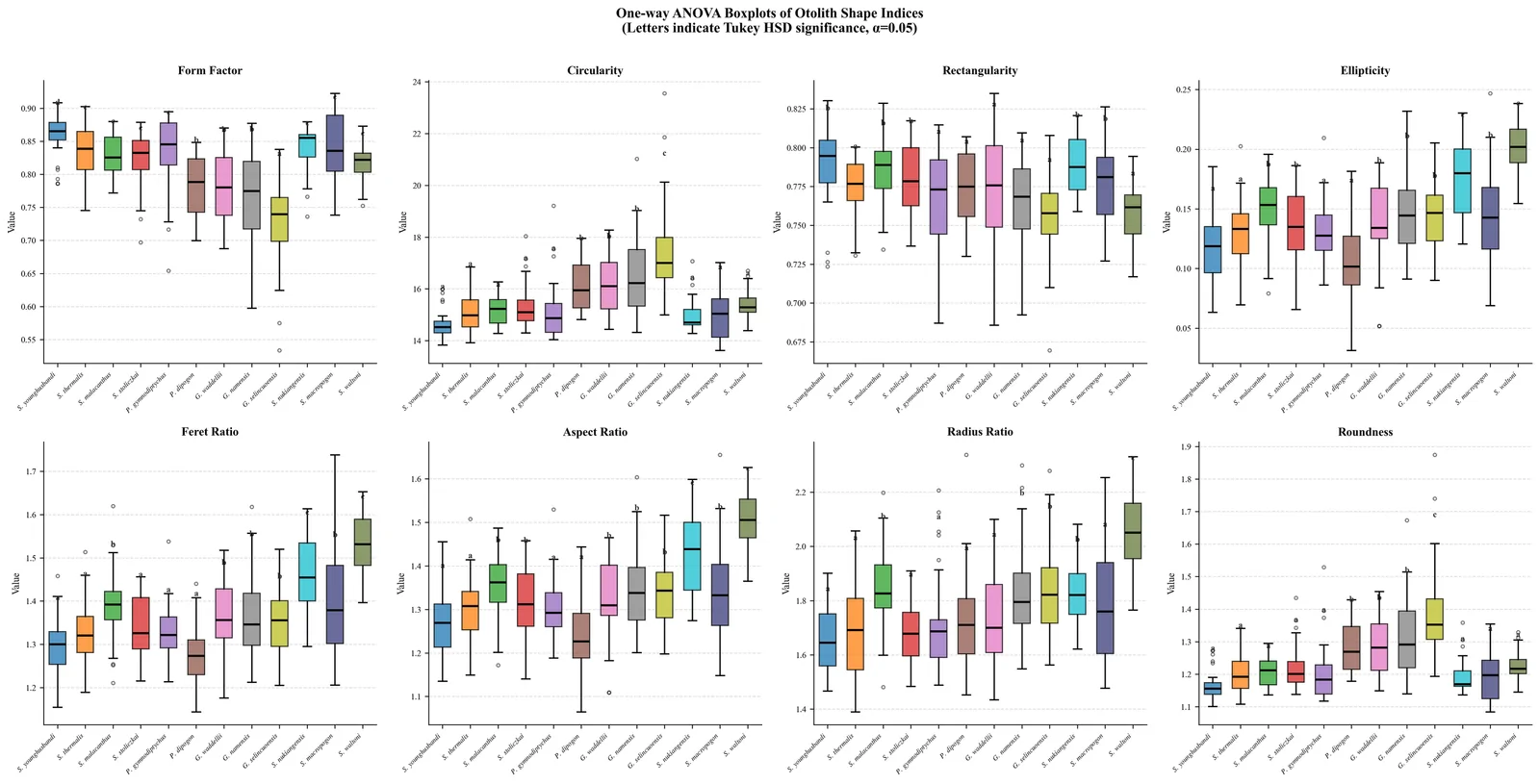
Boxplots of eight otolith shape indices for 12 Schizothoracinae species. Note: Lowercase letters above upper whiskers indicate significant differences from post-hoc multiple comparison tests. Groups sharing the same letter are not significantly different (*p* > 0.05), while groups with different letters differ significantly (*p* < 0.05). Open circles represent outliers.

The boxplots (Figure 4) intuitively display the data distribution characteristics of the 12 Schizothoracinae species across eight shape indices. Overall, the intergroup differentiation of ellipticity, aspect ratio, and Feret ratio was the most obvious, with *Schizothorax lhasaensis* located at the highest level in the aforementioned indices and showing little overlap with other species; the distribution of roundness and circularity among species was relatively concentrated, with a relatively low degree of interspecific differentiation, consistent with the ANOVA results.

#### 3.1.2. Principal Component Analysis of Shape Indices

**Figure 5:**
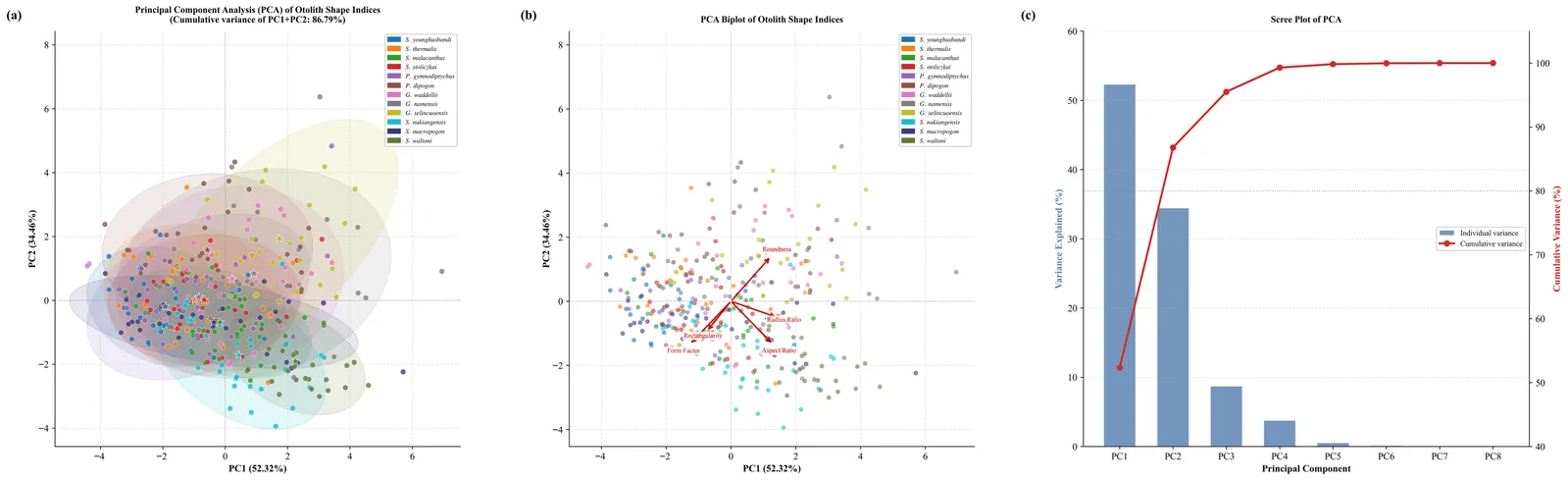
Results of principal component analysis for 12 Schizothoracinae species: (**a**) PC1–PC2 scatter plot with 95% confidence ellipses, (**b**) loading biplot, (**c**) Scree plot. Note: Solid coloured circles represent individual otolith samples; star markers denote the group centroid of each species.

Principal component analysis (PCA) was performed on the eight otolith shape indices of 12 Schizothoracinae species, extracting two principal components (PC1 = 4.197, PC2 = 2.765), with a cumulative variance contribution rate of 86.79% (Table 4, Figure 5a). The scree plot (Figure 5c) showed that eigenvalues dropped significantly after the first two principal components, further supporting the rationality of extracting two principal components.

The loading matrix showed (Table 4, Figure 5b) that PC1 was mainly positively contributed by radius ratio (0.408), aspect ratio (0.369), ellipticity (0.368), and Feret ratio (0.366), and negatively contributed mainly by form factor (−0.364), representing the elongation degree of the otolith; higher PC1 scores indicate greater differences between major and minor axes and a shape approaching a slender ellipse. PC2 was mainly positively contributed by circularity (0.401) and roundness (0.401), reflecting the regularity and complexity of the otolith contour; higher PC2 scores indicate a more complex otolith margin, larger perimeter, and more prominent marginal projections.

The principal component scatter plot (Figure 5a) showed that the 12 Schizothoracinae species overlapped to varying degrees in the 2D principal component space, but the 95% confidence ellipses of some species were clearly separated. *Schizothorax lhasaensis* shifted significantly along the positive direction of PC1, corresponding to its highest ellipticity, Feret ratio, and aspect ratio, clearly reflecting its slender otolith characteristics in the principal component space; *Ptychobarbus dipogon* distributed along the negative direction of PC1, with otoliths being the most rounded, short, and wide; *Gymnocypris* selincuoensis shifted along the positive direction of PC2, corresponding to its highest circularity and roundness, possessing the highest marginal complexity; the three species of *Gymnocypris* formed a relatively concentrated cluster in the principal component space, partially overlapping with *Schizopygopsis* and *Schizothorax*, indicating high conservatism of otolith morphology within *Gymnocypris*.

#### 3.1.3. t-SNE Dimensionality Reduction Visualization

**Figure 6:**
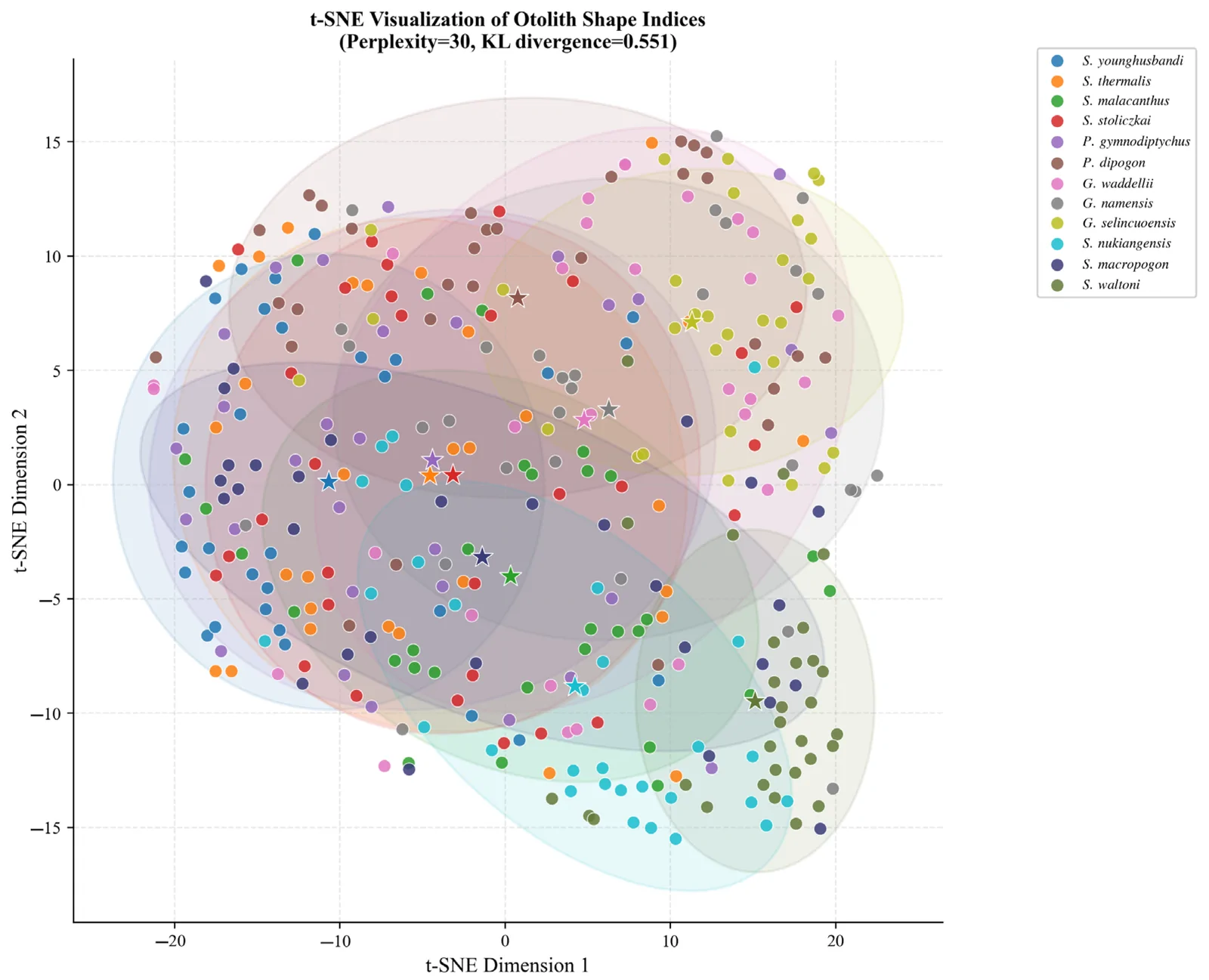
t-SNE visualization of otolith shape indices for 12 Schizothoracinae species. Note: Solid coloured circles represent individual otolith samples; star markers denote the group centroid of each species.

To further validate the PCA results and capture potential local non-linear structures, t-SNE dimensionality reduction was performed on the 8-dimensional shape features (Figure 6). The t-SNE results showed that the distribution pattern of individuals of the 12 Schizothoracinae species in 2D space was basic consistent with the PCA scatter plot: *Schizothorax lhasaensis* formed a relatively independent cluster in t-SNE space, confirming its extreme position in PCA; *Ptychobarbus dipogon* and *Ptychobarbus kaznakovi* distributed close to each other in space, suggesting high intrageneric morphological similarity; individuals of the three species of *Gymnocypris* and four species of *Schizopygopsis* overlapped extensively in t-SNE space, further confirming the high similarity of otolith morphology between these two genera; individuals of *Schizothorax macropogon* were extremely dispersed without an obvious clustering center, highly consistent with its extremely low correct classification rate (3.3%) in discriminant analysis.

The t-SNE results were highly consistent with the PCA conclusions, further confirming that the degree of interspecific differentiation in otolith morphology of the 12 Schizothoracinae species is limited, with large morphological overlap among closely related species, while also revealing the extreme specialization trend of a few species such as *Schizothorax lhasaensis* in morphospace.

#### 3.1.4. Discriminant Analysis of Shape Indices

Fisher linear discriminant functions were used to perform discriminant analysis with the eight otolith shape indices as independent variables and the 12 Schizothoracinae species as grouping variables. Due to high collinearity between circularity and roundness, SPSS automatically excluded circularity during stepwise discrimination, and the remaining seven indices were incorporated into the discriminant model. Eigenvalue analysis showed that the cumulative contribution rate of the first two discriminant functions was 76.5%; Wilks’ Lambda test indicated that all seven discriminant functions reached significant levels (*p* < 0.05), establishing that the constructed discriminant model is statistically significant.

Classification function coefficients are shown in Table 5. Substituting the seven otolith shape indices of each sample into the 12 discriminant equations, the score of each equation was calculated, and the category with the maximum score was designated as the predicted fish species for that sample.

**Figure 7:**
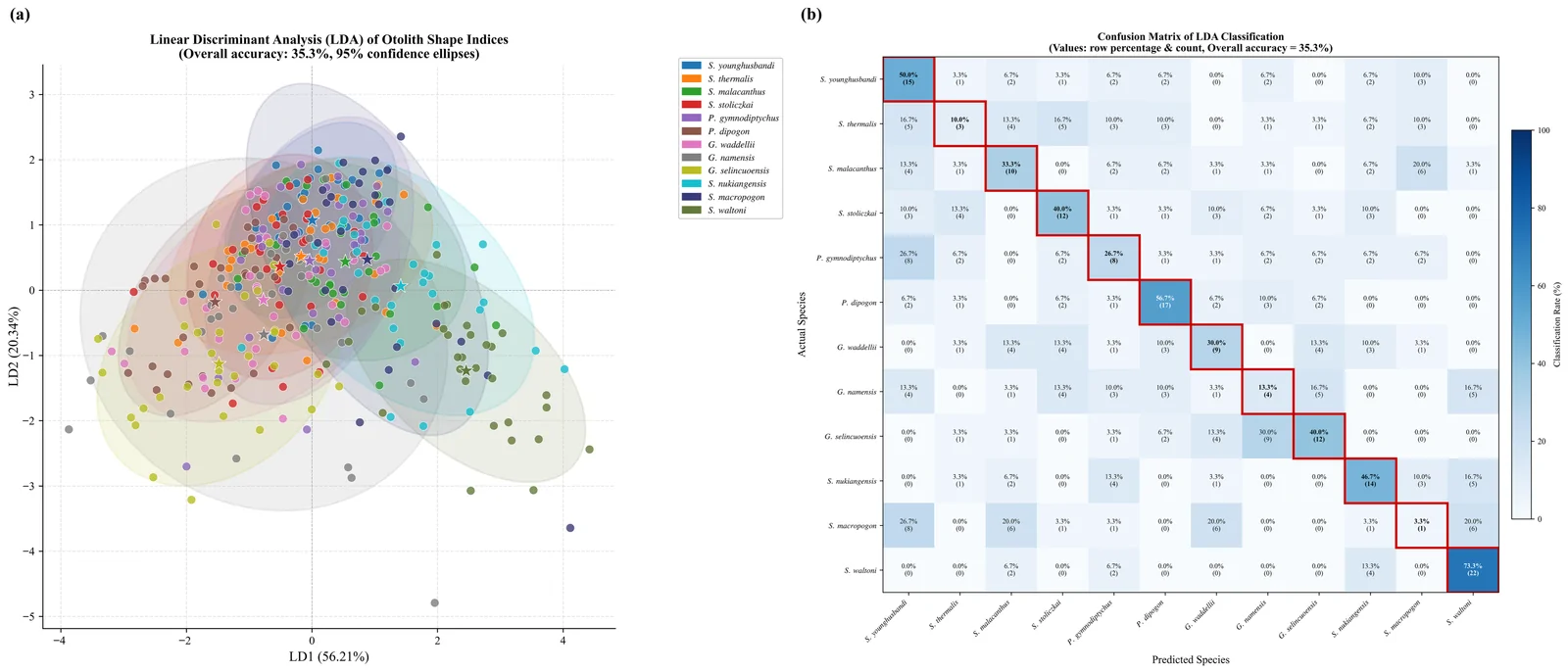
Combined plot of discriminant analysis for 12 Schizothoracinae species: (**a**) scatter plot based on the first two discriminant function values, (**b**) confusion matrix heatmap. Note: Solid coloured circles represent individual otolith samples; star markers denote the group centroid of each species; shaded ellipses show 95% confidence regions.

Original classification results indicated (Figure 7b) that the comprehensive correct discrimination rate of the 12 Schizothoracinae species was 35.3%, far higher than the random discrimination rate (8.33%), proving that otolith shape indices possess significant discriminant efficacy among species. Among them, *Schizothorax lhasaensis* achieved the highest correct discrimination rate (73.3%), with its otolith shape displaying the most unique interspecific differentiation features; *Ptychobarbus dipogon* (56.7%) and *Schizopygopsis lhasaensis* (50.0%) followed; *Schizothorax macropogon* had the lowest correct discrimination rate (3.3%), with a large number of individuals misclassified as *Schizopygopsis lhasaensis* (26.7%), *Schizopygopsis malacanthus* (20.0%), and *Gymnocypris waddellii* (20.0%), indicating that otolith morphology of *Schizothorax macropogon* overlaps heavily with other species; *Schizopygopsis thermalis* (10.0%) and *Gymnocypris namensis* (13.3%) also had correct rates at extremely low levels, with their individuals mainly misclassified as closely related species of the same genus or same water system.

At the genus level, the correct classification rate of *Schizothorax* (46.7%~73.3%) was significantly higher than that of *Schizopygopsis* (10.0%~50.0%) and *Gymnocypris* (13.3%~40.0%), indicating that interspecific differentiation of otolith morphology within *Schizothorax* is relatively high, whereas interspecific morphological overlap within *Schizopygopsis* and *Gymnocypris* is more severe. The discriminant scatter plot (Figure 7a) showed large-area overlaps among the 12 Schizothoracinae species in discriminant space: individuals of the four species of *Schizopygopsis* were concentrated in the center of the plot; *Schizothorax lhasaensis* shifted significantly toward the positive direction of Function 1 as a whole; *Ptychobarbus dipogon* showed dispersion along the positive direction of Function 2; individuals of *Schizothorax macropogon* were scattered without a clear clustering center.

### 3.2. Elliptic Fourier Method

#### 3.2.1. Otolith Contour Reconstruction

**Figure 8:**
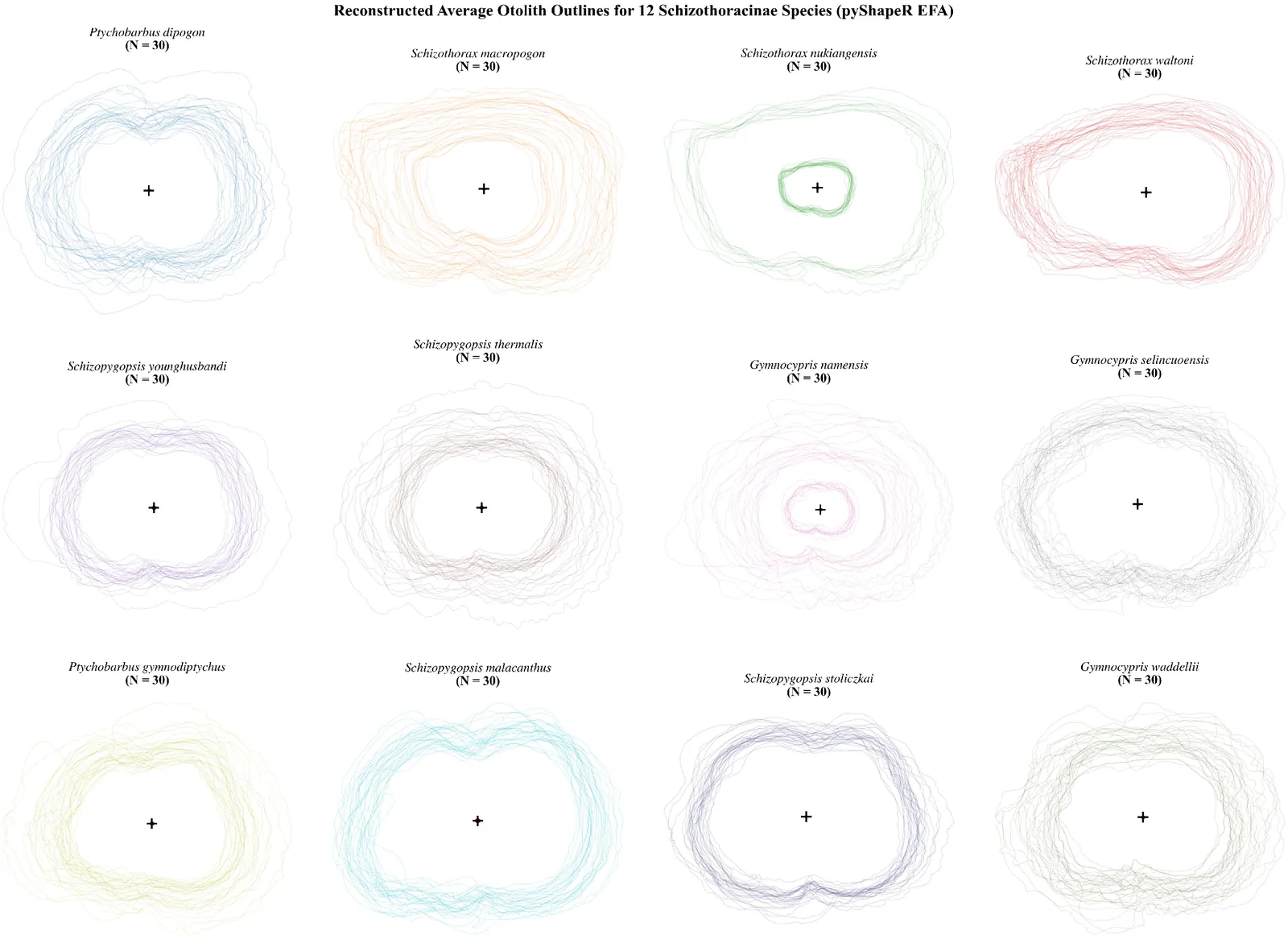
Reconstructed otolith outlines for 12 Schizothoracinae species. Note: Coloured lines represent individual otolith contours; the cross symbol indicates the centroid of the mean otolith contour for each species.

Extracting the first 15 harmonic orders of Elliptic Fourier harmonics (*n* = 15), the otolith contours of each species were mathematically reconstructed via Inverse Elliptic Fourier Analysis (Figure 8). From the reconstructed mean otolith contours, it can be seen that otoliths of the 12 Schizothoracinae species overall range from oval to elongated elliptical, with interspecific differences mainly reflected in the major-to-minor axis ratio, degree of dorsal protuberance, and sharpness of anterior and posterior ends. Among them, *Schizothorax* otolith contours are relatively slender with distinct anterior and posterior extensions, especially Schizothorax lhasaensis, whose otolith is the narrowest and most elongated, with a prominently pointed posterior end—consistent with its higher aspect ratio (1.506) and ellipticity (0.201) in the shape index method.

*Schizopygopsis*: otolith contours are relatively short and wide, with a relatively flat dorsal margin and rounded anterior end, displaying conservative overall morphology and small intrageneric differences. *Gymnocypris*: otolith contours are intermediate between the former two, presenting a regular oval shape with smooth margins and no obvious serrated projections. Ptychobarbus: otolith contours are compact and rounded with small major-to-minor axis differences, among which the otolith of *Ptychobarbus dipogon* is the most rounded and blunt among all species, having the lowest aspect ratio (1.247).

#### 3.2.2. Principal Component Analysis of Elliptic Fourier Coefficients

Principal component analysis was performed on the 60 normalized Elliptic Fourier coefficients, extracting 15 principal components with eigenvalues greater than 1, with a cumulative contribution rate of 60.44% (Table 6), among which the first three principal components had the highest contribution rates, being 6.92% (PC1), 6.28% (PC2), and 5.53% (PC3), respectively.

**Figure 9:**
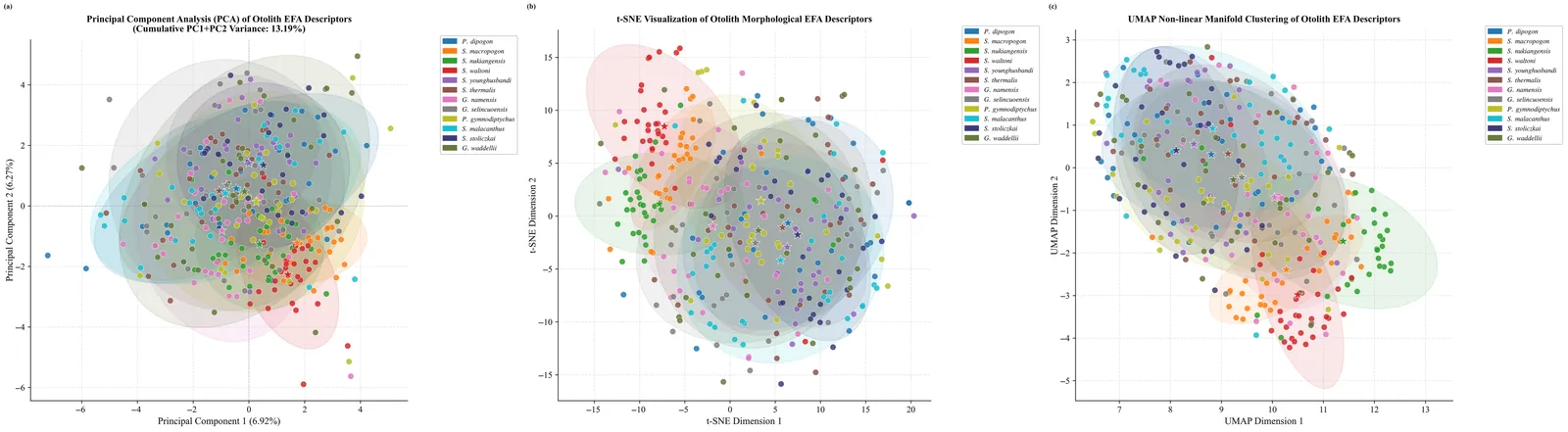
Dimensionality reduction results of EFA coefficients for 12 Schizothoracinae species. (**a**) PCA (**b**) t-SNE (**c**) UMAP. Note: Solid coloured circles represent individual otolith samples; star markers denote the group centroid of each species; shaded ellipses show 95% confidence regions.

The PC1-PC2 score scatter plot (Figure 9a) showed that the 12 Schizothoracinae species achieved a certain degree of separation in 2D principal component space, but some species still overlapped. *Schizothorax lhasaensis*, *Schizothorax nukiangensis*, and *Schizothorax macropogon* were distributed along the positive direction of PC1, corresponding to their relatively slender otolith contours; *Ptychobarbus dipogon* and *Ptychobarbus kaznakovi* were distributed along the negative direction of PC1, with relatively rounded otolith contours; the three species of *Gymnocypris* and four species of *Schizopygopsis* interwove in the central region of the plot, indicating large morphological overlap in otolith contours between these two genera.

The t-SNE (Figure 9b) and UMAP (Figure 9c) non-linear dimensionality reduction results were broadly consistent with the PCA scatter plot, but non-linear algorithms amplified local morphological differences; individuals of *Schizothorax* showed higher aggregation, with *Schizothorax lhasaensis*, *Schizothorax macropogon*, and *Schizothorax nukiangensis* forming relatively independent local clusters; *Ptychobarbus dipogon* and *Ptychobarbus kaznakovi* also displayed clearer local distribution features. Overall, all three dimensionality reduction methods demonstrated that *Schizothorax* and Ptychobarbus achieved higher separation in morphospace, while *Gymnocypris* and *Schizopygopsis* exhibited extensive morphological overlap, consistent with the conclusions of the shape index method.

#### 3.2.3. Discriminant Analysis of Elliptic Fourier Coefficients

Based on Fisher linear discriminant functions, discriminant analysis was conducted using 60 Elliptic Fourier coefficients as independent variables and 12 Schizothoracinae species as grouping variables. Eigenvalue analysis showed that the cumulative contribution rate of the first two discriminant functions was 62.30%; Wilks’ Lambda test indicated that all 11 discriminant functions reached significant levels (*p* < 0.05), establishing that the constructed discriminant model is statistically significant.

**Figure 10:**
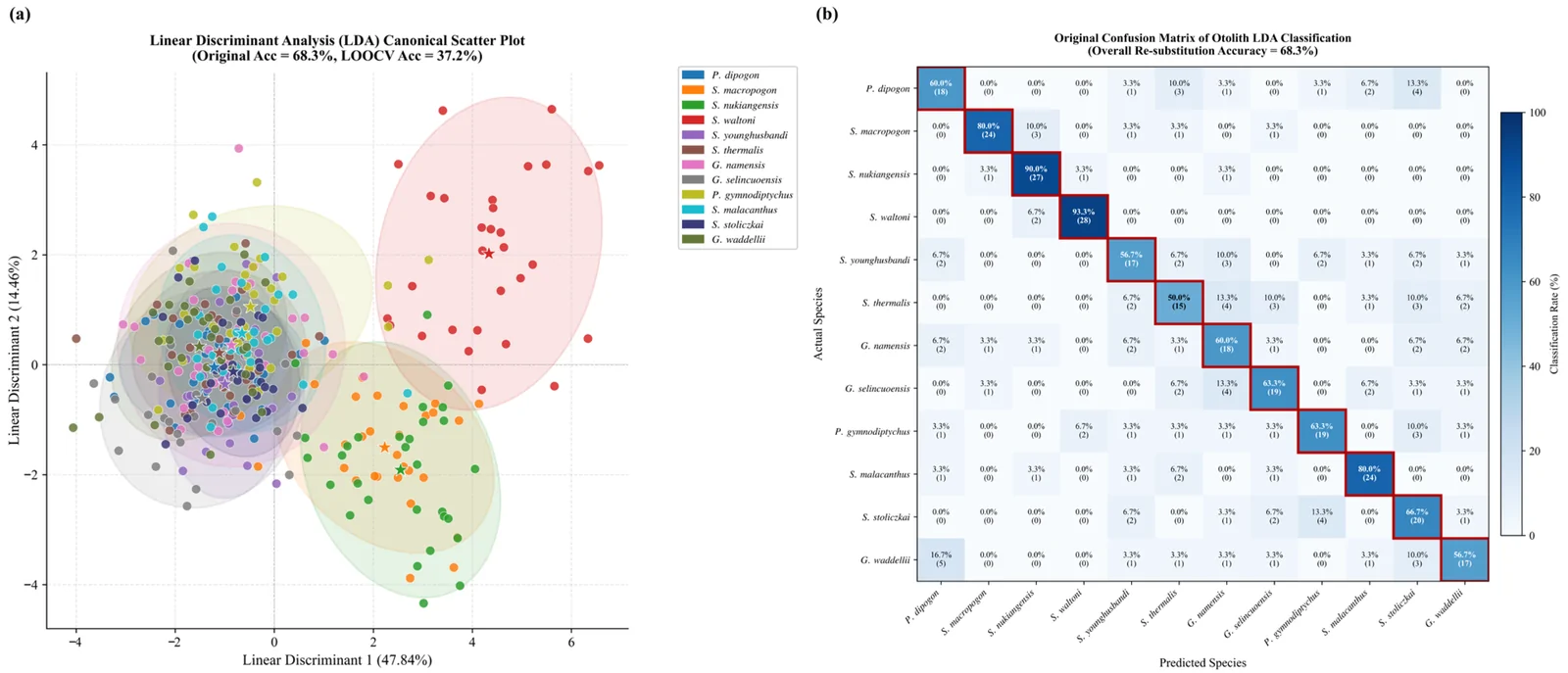
Discriminant analysis results of otolith EFA coefficients for 12 Schizothoracinae species: (**a**) scatter plot based on the first two discriminant function values, (**b**) confusion matrix heatmap. Note: Solid coloured circles represent individual otolith samples; star markers denote the group centroid of each species; shaded ellipses show 95% confidence regions.

Original classification results are shown in Figure 10; the comprehensive correct discrimination rate of the 12 Schizothoracinae species was 68.3%, nearly double that of the shape index method, indicating that Elliptic Fourier coefficients can more comprehensively capture interspecific difference information of otolith contours.

Among the 12 species, *Schizothorax lhasaensis* achieved the highest correct discrimination rate (93.3%), whose slender contour with a pointed posterior end possesses extremely high species specificity; *Schizothorax nukiangensis* ranked second (90.0%); correct discrimination rates of *Schizothorax macropogon* (80.0%) and *Schizopygopsis malacanthus* (80.0%) were also at relatively high levels. *Schizopygopsis thermalis* had the lowest correct discrimination rate, with its individuals mainly misclassified as *Gymnocypris namensis* (13.3%) and *Gymnocypris* selincuoensis (10.0%); correct discrimination rates of *Schizopygopsis lhasaensis* (56.7%) and *Gymnocypris waddellii* (56.7%) were also relatively low.

At the genus level, the original correct classification rate of *Schizothorax* was the highest (80.0–93.3%), followed by *Schizopygopsis* (50.0–80.0%), and *Gymnocypris* was relatively low (56.7–63.3%). The confusion matrix heatmap (Figure 10b) clearly demonstrated the main patterns of misclassification: misclassifications occurred mainly within *Gymnocypris* (*Gymnocypris namensis* vs. *Gymnocypris* selincuoensis; *Gymnocypris waddellii* vs. *Gymnocypris namensis*) and within *Schizopygopsis* (*Schizopygopsis lhasaensis* vs. *Schizopygopsis thermalis*), indicating that high similarity of otolith contours among closely related species is the primary reason for discrimination difficulty.

## 4. Discussion

### 4.1. Comparison and Evaluation of Two Otolith Shape Analysis Methods

In this study, the shape index method and Elliptic Fourier Analysis method were compared in 12 Tibetan Schizothoracinae fish species. Both methods exhibited significant differences in discriminant performance, information dimensionality, and application scenarios, which are closely related to their methodological essence.

The shape index method quantifies overall otolith contours using eight geometric shape parameters. Each parameter has explicit geometric meaning, facilitating biological interpretation, and the operational process has a high degree of standardization. In this study, principal component analysis of the eight shape parameters showed that the cumulative contribution rate of the first two principal components reached 86.79%, indicating that these eight parameters provide a good representation of the overall variation in otolith shape. However, the comprehensive correct LDA discrimination rate of this method was only 35.3%, significantly lower than general levels in multi-species comparative studies. This result is not surprising: shape indices extract limited information from continuous contours, capturing only gross proportions while losing substantial local details; on the other hand, this study involves 12 species belonging to four genera, and the substantial increase in intergroup category count makes the discrimination task itself far exceed similar studies involving only 2–4 species or geographic stocks. When comparing these classification accuracies with previous literature, it is crucial to consider the different numbers of species and varying analytical contexts. For instance, Wang et al. achieved an 81.65% comprehensive discrimination rate using 7 shape parameters in only three *Schizothorax* species in the Yarkand River [17]. Zhou found a 59.7% comprehensive discrimination rate for the shape index method across three geographic stocks of *Schizothorax grahami* in the Chishui River [16], indicating that discriminant performance of shape indices declines sharply as the number of groups increases. Studies by Song et al. on Sciaenidae [24] and Mesa et al. [25,26] also support the above pattern.

In contrast, EFA reconstructs closed otolith contours via Fourier series, applying 60 Fourier coefficients to subsequent principal component extraction and discriminant analysis, elevating the overall correct discrimination rate to 68.3%, representing a 33.0 percentage point increase over the shape index method. Notably, the shape variation in EFA coefficients was dispersed across the first 15 principal components with a cumulative contribution rate of 60.44%, far lower than the 86.79% of the first two principal components in the shape index method. This contrast reveals the fundamental difference between the two methods: Fourier coefficients capture multi-level morphological information of contours from overall trends to local microstructures via superposition of multiple frequency components. Furthermore, the remaining approximately 40% of the variance in the EFA components likely represents higher-frequency micro-structural variations, which could encapsulate both intrinsic biological individual differences and methodological background noise, thereby highlighting the complexity of capturing full otolith contours. However, it must be explicitly acknowledged that comparing an 8-dimensional scalar model (shape indices) with a 60-dimensional Fourier model contains inherent mathematical asymmetry, as higher-dimensional models naturally possess greater descriptive capacity. The morphological information captured by EFA is far more extensive than that of eight geometric parameters, thereby providing a more solid informational foundation for discriminant analysis [17,22,27]. Studies by Stransky et al. [26], Fang et al. [28], and Zhang et al. [29] in various fish species also demonstrated that higher overall discrimination success rates can be obtained using Elliptic Fourier coefficients. Tracey et al. [11] further pointed out that applying constrained canonical analysis can further enhance the discriminant performance of Fourier coefficients. In addition, the advantage of neural network methods significantly outperforming traditional statistical methods in otolith morphological discrimination [30] suggests that combining deep learning with EFA coefficients represents an important pathway for further improving discrimination accuracy.

In summary, in large-scale classification scenarios involving multiple genera and species, the EFA method is more applicable due to its higher information density and superior discriminant performance, making it the preferred method for species identification; while the shape index method, owing to its intuitive biological interpretability, still retains indispensable auxiliary value in descriptive analysis and ecological research of otolith morphology.

### 4.2. Genus-Level Divergence Patterns of Otolith Morphology in Schizothoracinae

Both analytical methods consistently revealed a clear gradient in otolith morphological differentiation among the three genera of Schizothoracinae: interspecific differentiation within *Schizothorax* was the highest, *Schizopygopsis* ranked second, and *Gymnocypris* exhibited the lowest internal differentiation, with extensive morphological overlap existing between the latter two genera. This pattern provides a new perspective for understanding the adaptive radiation and morphological evolution of Schizothoracinae in the Qinghai–Xizang Plateau.

The higher degree of morphological differentiation in *Schizothorax* is closely related to its unique biogeographical distribution pattern and ecological niche diversity. *Schizothorax* is the most species-rich genus in Schizothoracinae, distributed across different river reaches, mainstreams, and tributaries of major water systems in the Qinghai–Xizang Plateau [2], with significant interspecific differentiation across ecological dimensions such as water current velocity, substrate type, food resources, and inhabited water depth. Existing research indicates that flow velocity and substrate type significantly influence otolith morphology. Otoliths from rapid-current habitats tend to be compact and elongated, whereas those from sluggish or standing waters are more rounded [7]. In this study, otoliths of *Schizothorax lhasaensis* were significantly higher than those of other species in ellipticity, aspect ratio, and Feret ratio, with overall otolith contours displaying a slender and pointed shape, closely matching its ecological habit of preferring torrent river reaches. This supports the hypothesis proposed by previous ecological literature that riverine habitats are often associated with elongated otoliths, though direct environmental causality remains to be tested. In the EFA method, its correct discrimination rate reached up to 93.3%, also confirming that its otolith contour possesses extremely high species specificity among the 12 study species. Furthermore, the significant power function relationship between otolith morphology and standard body length (R^2^ between 0.409 and 0.749) further suggests a tight coupling relationship between otolith growth trajectory and fish body growth pattern [17]; differences in growth rate and growth mode among different ecotype fishes will be synchronously reflected in otolith contour morphology. However, we also acknowledge the possibility that the higher degree of morphological differentiation observed in Schizothorax may partly reflect the specific geographic sample structure and uneven species distribution within our study, which requires further validation with broader sampling.

*Gymnocypris* and *Schizopygopsis* exhibited low degrees of otolith morphological differentiation, displaying extensive intrageneric and intergeneric morphological overlap in both analysis methods. This mutually validates findings at the genomic level that no significant differences exist between *Gymnocypris* and *Schizopygopsis* [31], demonstrating that the low level of genetic differentiation between these two genera is likewise reflected in their otolith phenotypic traits. Notably, the otolith form factor of the three species of *Gymnocypris* was significantly lower than that of other genera (*p* < 0.05), exhibiting more rounded and smooth contour features. Multiple studies across different taxa have documented a ‘rounding effect’ of lacustrine and slow-flowing habitats on otolith morphology [7,8], and the results of this study highly coincide with the ecological habits of these three species, mainly distributed in Tibetan Plateau lakes and large sluggish river-lake junction areas [3]. This finding suggests that in Schizothoracinae, otolith roundness exhibits a strong association with lacustrine habitats. However, we acknowledge that the present dataset alone does not fully validate its performance as a universal biomarker, and broader systematic testing across different ecological gradients is required.

### 4.3. Morphological Discrimination Potential and Limitations of Otolith Morphology in Schizothoracinae

The analysis results of this study indicate that otolith morphology possesses clear potential for morphological discrimination in Schizothoracinae. However, while a 68.3% overall classification accuracy provides valuable insights into ecological and morphological adaptations, it is insufficient to serve as a standalone diagnostic tool for strict taxonomic identification. Furthermore, its discrimination efficacy among closely related species is constrained by multiple factors, and its limitations cannot be ignored.

Regarding discrimination potential, one-way ANOVA results for both shape index and EFA methods showed that the 12 Schizothoracinae species exhibited highly significant differences across all eight shape parameters and 60 Fourier coefficients, proving at the population statistical level that otolith morphology contains rich species-specific information. In the EFA method, correct discrimination rates for *Schizothorax lhasaensis* (93.3%) and *Schizothorax nukiangensis* (90.0%) reached the practical accuracy threshold for fishery resource identification, and correct rates for *Schizothorax macropogon* and *Schizopygopsis malacanthus* (both 80.0%) were also at relatively high levels. This demonstrates that for species with unique otolith morphology, the EFA method holds practical promotion value in scenarios such as origin identification, market regulation, and fishery administration enforcement. This study is the first to systematically apply otolith morphometrics to 12 Schizothoracinae species across four genera in Tibet, establishing an otolith morphometric feature database for each species at the genus and partial species levels, providing reference morphological evidence for phenotypic differentiation and ecological monitoring of endemic fishes in the Qinghai–Xizang Plateau, though it cannot serve as a standalone tool for taxonomic revision.

Regarding methodological limitations, high misclassification rates among closely related species (especially within *Gymnocypris* and within *Schizopygopsis*) reveal essential limitations of 2D otolith contour analysis methods. The generation of this limitation stems from several levels of reasons: first, interspecific differences in otolith morphology are constrained by genetic foundations; for closely related species with inherently low genetic differentiation (such as *Gymnocypris* and *Schizopygopsis* [31]), the upper limit of their otolith phenotypic differentiation is constrained by genetic homogeneity; second, extreme habitat pressures such as high altitude, hypoxia, low temperature, and strong radiation on the Qinghai–Xizang Plateau exert strong convergent selection on phenotypes of Schizothoracinae, which may cause otoliths of different species to undergo phenotypic convergence during high-altitude adaptive evolution, thereby increasing the difficulty of morphological discrimination; third, the amount of information captured by 2D contour analysis itself is limited; 3D spatial structures, internal texture features (such as annual rings, core and microstructures), and otolith microchemical composition (element fingerprints) of otoliths all carry substantial species and stock identity information, none of which can be obtained through 2D contour analysis. Notably, in a study by Zhou et al. [32] on *Notothenia rossii*, wavelet coefficients were able to detect significant morphological differences between two regional stocks while traditional shape parameters showed no significance, suggesting that algorithmic differences in capturing high-frequency local contour features significantly affect discrimination efficacy for closely related taxa, and combined application of multiple contour analysis methods may improve resolution for closely related species.

Furthermore, regarding statistical limitations, the Leave-One-Out Cross-Validation (LOOCV) used in our LDA models may produce slightly optimistic accuracy estimates compared to independent external validation. Future studies with larger sample sizes should employ K-fold cross-validation or independent external test cohorts to more robustly evaluate the model’s generalizability across different geographical populations.

In response to the above limitations, future research can seek breakthroughs along the following pathways: combining 2D otolith contour analysis with otolith microchemistry (such as Sr/Ca, Ba/Ca element ratios) and stable isotope techniques to construct a multi-index integrated species identification system; integrating contour analysis with molecular markers to clarify the genetic basis of otolith morphological differentiation from a genetic-phenotype association perspective; introducing deep learning for end-to-end feature learning on otolith contour images, which is expected to automatically discover local morphological differences difficult to capture by traditional Fourier analysis, further improving discrimination accuracy among closely related species; with the popularization of 3D micro-CT technology, otolith research based on 3D geometric morphometrics is expected to provide quantitative information in a brand-new dimension for species identification and ecological research of Schizothoracinae.

## 5. Conclusions

This study took 12 Schizothoracinae fish species in Tibet as research objects, comparing the application effects of the shape index method and Elliptic Fourier Analysis method in interspecific otolith discrimination. In the shape index method, 12 species exhibited highly significant differences across all eight shape indices, while the comprehensive correct LDA discrimination rate reached 68.3% for EFA, an increase of 33.0 percentage points compared to the shape index method. Both methods showed that the degree of otolith morphological differentiation was highest in *Schizothorax*, while extensive morphological overlap existed between *Gymnocypris* and *Schizopygopsis*. Elliptic Fourier Analysis can capture fine morphological differences in otolith contours more effectively, serving as an effective tool for phenotypic differentiation of Schizothoracinae. However, it must be emphasized that the performance of EFA varies substantially among species; while otolith morphology possesses strong potential for discrimination at the genus and certain species levels, the discrimination efficacy among closely related lineages remains highly limited.

This study analyzed only two-dimensional contour features of otoliths, failing to cover three-dimensional structures and internal microchemical features of otoliths. Future research can combine otolith morphological analysis with molecular markers or otolith microchemistry to construct a multi-level species discrimination system. With the rapid development of three-dimensional imaging technology and machine learning methods, otolith morphological analysis based on three-dimensional spatial scales is expected to provide more precise scientific evidence for species identification and resource protection of Schizothoracinae.

## Figures and Tables

**Table 1:** Basic information about the sampling sites and 12 Schizothoracinae species samples.

Species	Site	Number	Longitude	Latitude	*n*	Total Length Ranges (cm)
*S. younghusbandi*	Bobu Qu	a	92.34195543	31.49648596	30	12.6–27.2
*S. thermalis*	Tangra Yumco	b	86.54891311	30.487896	7	24.4–35.3
	Zhunbu Zangbo	c	89.079691	30.80640537	23	15.0–27.5
*S. malacanthus*	Zi Qu, Tongpu Township, Jinsha River	d	98.342488	31.580435	24	13.8–20.4
	Confluence of Dairen Nong Qu and Jinsha River	e	97.793983	32.515968	6	14.4–16.6
*S. stoliczkai*	Shiquan River	f	80.46721458	32.53997573	30	18.4–20.9
*G. waddellii*	Yamdrok Lake	g	90.53224	28.779989	30	22.3–43.2
*G. namensis*	Lake Namtso	h	90.25298889	30.89393333	30	9.6–40.9
*G. selincuoensis*	Confluence of Ngari Zangbo and Siling Co	i	88.574694	31.749556	15	25.8–37.5
	Confluence of Zhagen Zangbo and Siling Co	j	88.522939	31.819669	15	20.7–36.4
*P. gymnodiptychus*	Gaiqu Mianda Township	k	97.188719	31.987167	30	13.3–33.5
*P. dipogon*	Duobu	l	92.48241	29.729055	30	10.2–28.9
*S. macropogon*	Luxia	m	94.680691	29.455555	30	8.4–39.5
*S. waltoni*	Jiacha	n	92.48241	29.23423	30	13.3–32.6
*S. nukiangensis*	Jiangda Township Section, Nujiang River	o	94.40629798	31.48158574	11	21.5–26.9
	Biru Section, Nujiang River	p	93.74945492	31.47748033	15	20.5–32.2
	Confluence of Nujiang River and Tongna Qu	q	95.76432138	30.93467392	4	21.3–25.7

Note: The sampling point numbers in the table correspond to Figure 1, as shown below.

**Table 2:** Measurement parameters and shape indices of otolith.

Size Parameters	Shape Indices
Otolith area(A)	Roundness = 4A/πFL^2^
Otolith perimeter(P)	Format-factor = 4πA/P^2^
Otolith length(FL)	Circularity = P^2^/A
Otolith width(FW)	Rectangularity = A/(FL × FW)
Maximum Feret diameter(F_max_)	Ellipticity = (FL − FW)/(FL + FW)
Minimum Feret diameter(F_min_)	Radius ratio = R_max_/R_min_
Maximum radius(R_max_)	Feret ratio = F_max_/F_min_
Minimum radius(R_min_)	Aspect ratio = FL/FW

**Table 3:** One-way ANOVA results of otolith shape indices for 12 Schizothoracinae species.

Species	Form Factor	Circularity	Rectangularity	Ellipticity	Feret Ratio	Aspect Ratio	Radius Ratio	Roundness
*S. younghusbandi*	0.859 ± 0.033 ^d^	14.645 ± 0.586 ^a^	0.788 ± 0.027 ^b^	0.118 ± 0.030 ^a^	1.297 ± 0.068 ^a^	1.270 ± 0.077 ^a^	1.653 ± 0.112 ^a^	1.165 ± 0.047 ^a^
*S. thermalis*	0.834 ± 0.043 ^c^	15.111 ± 0.812 ^a^	0.775 ± 0.019 ^a^	0.131 ± 0.030 ^a^	1.327 ± 0.080 ^a^	1.303 ± 0.079 ^a^	1.699 ± 0.174 ^a^	1.203 ± 0.065 ^a^
*S. malacanthus*	0.830 ± 0.030 ^c^	15.157 ± 0.552 ^a^	0.784 ± 0.021 ^b^	0.148 ± 0.029 ^b^	1.390 ± 0.087 ^b^	1.349 ± 0.078 ^b^	1.839 ± 0.159 ^b^	1.206 ± 0.044 ^a^
*S. stoliczkai*	0.821 ± 0.043 ^c^	15.353 ± 0.869 ^a^	0.782 ± 0.023 ^b^	0.138 ± 0.030 ^b^	1.340 ± 0.071 ^a^	1.322 ± 0.080 ^b^	1.684 ± 0.116 ^a^	1.222 ± 0.069 ^a^
*P. kaznakovi*	0.833 ± 0.056 ^c^	15.161 ± 1.142 ^a^	0.769 ± 0.031 ^a^	0.130 ± 0.028 ^a^	1.327 ± 0.067 ^a^	1.302 ± 0.076 ^a^	1.721 ± 0.188 ^a^	1.206 ± 0.091 ^a^
*P. dipogon*	0.780 ± 0.048 ^b^	16.162 ± 1.004 ^b^	0.774 ± 0.021 ^a^	0.109 ± 0.036 ^a^	1.279 ± 0.073 ^a^	1.247 ± 0.091 ^a^	1.730 ± 0.182 ^a^	1.286 ± 0.080 ^b^
*G. waddellii*	0.783 ± 0.055 ^b^	16.124 ± 1.145 ^b^	0.775 ± 0.035 ^a^	0.137 ± 0.038 ^b^	1.364 ± 0.084 ^b^	1.322 ± 0.099 ^b^	1.739 ± 0.181 ^a^	1.283 ± 0.091 ^b^
*G. namensis*	0.770 ± 0.068 ^b^	16.463 ± 1.563 ^b^	0.766 ± 0.026 ^a^	0.145 ± 0.037 ^b^	1.362 ± 0.101 ^b^	1.345 ± 0.104 ^b^	1.834 ± 0.192 ^b^	1.310 ± 0.124 ^b^
*G. selincuoensis*	0.727 ± 0.069 ^a^	17.463 ± 1.879 ^c^	0.755 ± 0.026 ^a^	0.142 ± 0.028 ^b^	1.355 ± 0.072 ^b^	1.334 ± 0.076 ^b^	1.841 ± 0.161 ^b^	1.390 ± 0.150 ^c^
*S. nukiangensis*	0.839 ± 0.035 ^c^	14.997 ± 0.661 ^a^	0.788 ± 0.019 ^b^	0.177 ± 0.034 ^c^	1.462 ± 0.090 ^c^	1.434 ± 0.100 ^c^	1.824 ± 0.114 ^b^	1.193 ± 0.053 ^a^
*S. macropogon*	0.839 ± 0.054 ^c^	15.042 ± 0.981 ^a^	0.777 ± 0.026 ^b^	0.146 ± 0.040 ^b^	1.395 ± 0.115 ^b^	1.348 ± 0.113 ^b^	1.772 ± 0.199 ^a^	1.197 ± 0.078 ^a^
*S. waltoni*	0.816 ± 0.028 ^c^	15.415 ± 0.537 ^a^	0.757 ± 0.019 ^a^	0.201 ± 0.022 ^c^	1.532 ± 0.067 ^c^	1.506 ± 0.068 ^c^	2.052 ± 0.162 ^c^	1.227 ± 0.043 ^a^
F Value	18.301	17.448	5.713	18.111	21.624	19.054	12.677	17.448
*p* Value	<0.001	<0.001	<0.001	<0.001	<0.001	<0.001	<0.001	<0.001

Note: Data in the table are mean ± standard deviation. Superscript letters indicate significant differences among different species at the *p* = 0.05 confidence level; the same letter indicates no significant difference, and different letters indicate significant differences. Multiple comparisons used the Tukey HSD test.

**Table 4:** Loadings and eigenvalues of principal component analysis for otolith shape indices of 12 Schizothoracinae species.

Shape Indices	Principal Components	
	PC1	PC2
Roundness	0.353	0.401
Format factor	−0.364	−0.385
Circularity	0.353	0.401
Rectangularity	−0.214	−0.268
Ellipticity	0.368	−0.383
Radius ratio	0.408	−0.144
Feret ratio	0.366	−0.380
Aspect ratio	0.369	−0.384
Eigenvalue	4.197	2.765
Variance explained (%)	52.32	34.46

**Table 5:** Classification function coefficients for 12 Schizothoracinae species.

Shape Index	*S. younghusbandi*	*S. thermalis*	*S. malacanthus*	*S. stoliczkai*	*P. kaznakovi*	*P. dipogon*	*G. waddellii*	*G. namensis*	*G. selincuoensis*	*S. nukiangensis*	*S. macropogon*	*S. waltoni*
Roundness	15,351.524	15,317.120	15,333.381	15,296.357	15,333.014	15,258.566	15,285.254	15,308.421	15,335.884	15,355.754	15,359.952	15,346.889
Format factor	27,300.040	27,232.577	27,268.637	27,182.502	27,264.806	27,095.917	27,145.629	27,189.633	27,213.213	27,311.233	27,318.526	27,316.571
Rectangularity	1380.971	1362.121	1383.224	1377.936	1351.046	1392.727	1386.316	1370.917	1370.249	1376.148	1363.589	1321.330
Ellipticity	−71,796.876	−71,669.147	−71,532.733	−71,643.161	−71,648.004	−72,036.676	−71,688.600	−71,754.530	−71,584.858	−71,896.400	−71,701.653	−72,285.501
Radius ratio	501.102	498.478	505.376	494.145	500.570	499.298	493.425	499.764	497.212	498.677	500.862	508.370
Feret ratio	339.296	336.955	374.751	331.925	336.822	324.698	366.671	322.999	326.921	369.477	387.960	359.862
Aspect ratio	26,121.845	26,082.291	25,998.002	26,081.794	26,073.601	26,210.311	26,064.434	26,124.718	26,058.326	26,156.001	26,054.814	26,307.366
Constant	−34,208.400	−34,053.342	−34,090.137	−33,987.847	−34,085.634	−33,998.604	−33,967.447	−34,042.649	−34,032.974	−34,288.016	−34,210.393	−34,392.660

Note: Discriminant function form is Yi = β_1_X_1_ + β_2_X_2_ + … + β_7_X_7_ + Ci, where X_1_–X_7_ are roundness, form factor, rectangularity, ellipticity, radius ratio, Feret ratio, and aspect ratio, respectively; Ci is a constant. Circularity was excluded due to high collinearity with roundness.

**Table 6:** Results of principal component analysis for EFA coefficients of 12 Schizothoracinae species.

Component	Eigenvalue	Variance (%)	Cumulative (%)
PC1	4.092	6.92	6.92
PC2	3.712	6.28	13.19
PC3	3.271	5.53	18.72
PC4	2.878	4.87	23.59
PC5	2.508	4.24	27.82
PC6	2.392	4.04	31.87
PC7	2.348	3.97	35.84
PC8	2.113	3.57	39.41
PC9	2.005	3.39	42.80
PC10	1.959	3.31	46.11
PC11	1.858	3.14	49.25
PC12	1.755	2.97	52.21
PC13	1.742	2.94	55.16
PC14	1.604	2.71	57.87
PC15	1.519	2.57	60.44

## Data Availability

The data presented in this study is available on request from the corresponding author.
